# Linking families with pre-school children from healthcare services to community resources: a systematic review protocol

**DOI:** 10.1186/s13643-017-0417-7

**Published:** 2017-03-08

**Authors:** Jacky Burns, David I. Conway, Wendy Gnich, Lorna M. D. Macpherson

**Affiliations:** 1 NHS Fife, Ward 8 Cameron Hospital, Leven, Fife KY8 5RG UK; 2 School of Medicine, Dentistry and Nursing, University of Glasgow, Leven, Fife UK

**Keywords:** Referral, Signposting, Linking, Social prescribing, Social determinants of health, Community, Children, Pre-school, Early years

## Abstract

**Background:**

Poor health and health inequalities persist despite increasing investment in health improvement programmes across high-income countries.

Evidence suggests that to reduce health inequalities, a range of activities targeted at different levels within society and throughout the life course should be employed. There is a particular focus on addressing inequalities in early years as this may influence the experience of health in adulthood.

To address the wider determinants of health at a community level, a key intervention which can be considered is supporting patients to access wider community resources. This can include processes such as signposting, referral and facilitation. There is a lack of evidence synthesis in relation to the most effective methods for linking individuals from health services to other services within communities, especially when considering interventions aimed at families with young children.

**Method/design:**

The aim of this study is to understand the way health services can best help parents, carers and families with pre-school children to engage with local services, groups and agencies to address their wider health and social needs. The review may inform future guidance to support families to address wider determinants of health.

The study is a systematic review, and papers will be identified from the following electronic databases: Web of Science, Embase, MEDLINE and CINAHL. A grey literature search will be conducted using an internet search engine and specific grey literature databases (TRiP, EThOS and Open Grey). Reference lists/bibliographies of selected papers will be searched. Quality will be assessed using the Effective Public Health Practice Project Quality Assessment Tool for quantitative studies and the CASP tool for qualitative studies. Data will be synthesised in a narrative form and weighted by study quality.

**Discussion:**

It is important to understand how health services can facilitate access to wider services for their patients to address the wider determinants of health. This may impact on the experience of health inequalities. This review focuses on how this can be achieved for families with pre-school children, and the evidence obtained will be useful for informing future guidance on this topic.

**Systematic review registration:**

PROSPERO CRD42016034066

**Electronic supplementary material:**

The online version of this article (doi:10.1186/s13643-017-0417-7) contains supplementary material, which is available to authorized users.

## Background

Tackling health inequalities is a key priority for those who hope to improve population health [1–3]. However, the health of a population is influenced by a number of different social factors, more commonly referred to as the social determinants of health [4]. Although healthcare services can address health issues, addressing the wider determinants of health can be very difficult and new ways of engaging patients to address these wider issues are important [2, 3, 5]. This is especially relevant when considering the impact health inequalities have on young children; therefore, addressing the needs of families with young children is a priority across many different regions [2, 6].

Many diseases are chronic and progressive in nature, with many conditions beginning to develop in childhood. Studies in high-income countries have demonstrated that health inequalities can be observed from a very young age [7, 8]. Often, health inequalities observed in childhood persist into adulthood [9, 10]. Based on these observations, a life-course approach has proven useful for understanding inequalities in health in different populations [11].

To tackle the effects of poor population health and large-scale inequalities in health, many countries have made investment in the prevention of disease. Nevertheless, despite the fiscal and infrastructure investment and improvement in health observed, significant inequalities in child health persist. Evidence from across several countries indicates that many of the most deprived individuals in the population continue to exhibit the highest levels of disease [12–14]. The inability to impact significantly on health inequalities through public health programmes may be due to the limited efficacy and reach of clinical prevention and health education interventions at reducing inequalities and a need to consider the wider determinants of health [15–17].

### Principles of reducing inequalities

A number of key publications reflect current knowledge on the principles of reducing inequalities. The World Health Organisation Commission on the Social Determinants of Health [18] proposes three key actions:

- Improving the daily conditions of life
- Tackling the inequitable distribution of power, money and resources
- Measuring and understanding the problem and addressing the impact of action

It approaches the issue of inequality from a social determinants perspective and gives the broad overarching actions which should be undertaken.

The Marmot Review [6] built upon the Commission on the Social Determinants of Health for a UK context and proposed the theory of proportionate universalism. This is where resources are made available to all, but with an intensity of support proportionate to the level of need. In this publication, the importance of early years’ interventions was also asserted.

The Working for Health Equity report [19] developed the idea of health services working with communities and engaging in partnerships for the mutual benefit of all. This theme was also reflected in the 2013 NHS Scotland report on health inequalities [20] which pulled together a number of key ideas, suggesting that multiple actions at different levels were required to address health inequalities at a population level. This is described as downstream, midstream and upstream activities.

Downstream activities are actions made close to the individual such as clinical preventive activities, whereas upstream actions are those distant from the individual and instituted at a population level, this might include legislative action such as taxation. The interaction at a community level is captured in midstream actions including community engagement and development [15].

Whilst upstream actions are predominantly driven by government programmes, and downstream actions are in the hands of individual practitioners and patients, midstream activities are those which can be designed, developed and actioned at a community level. There is a growing recognition that closer working between health and social care services and also with community, voluntary and third sector services is required to influence the experience of health inequalities in communities.

### Signposting and linking

To influence the wider determinants at the community level and improve links between health and social care services, it may be beneficial to facilitate access to existing community resources and engagement activities through the process of linking individuals to services. These linking activities could include signposting, referral or facilitation to engage with services or activities beyond traditional health services. This may allow healthcare teams to support individuals to address the wider determinants of health by engaging with social and community resources. Understanding the process of linking individuals to community services from a healthcare setting may allow for more effective use of existing community resources and activities and better utilisation of existing skills within clinical teams to influence health inequalities, yet there is a lack of evidence synthesis, particularly with regard to the best process for linking, in this topic area.

Current evidence relating to signposting is diverse and comes from different disciplines including social work, education and healthcare [21–25]. A range of terms can be used to describe the interventions including social prescribing, referral, signposting and linking.

A review related to “linking schemes” between healthcare and community resources for patients with chronic conditions was published in 2015 [26]. This review, which focussed on older adults living with a chronic condition and makes no reference to children, highlighted the disparate nature and lack of primary empirical evidence on this topic. It included many grey literature publications in the review as often these interventions are not reported in peer-reviewed journals but found in unpublished evaluation reports.

The key findings of the review highlighted the importance of having active involvement of healthcare practitioners in the process of linking and the essential role of facilitators for patients to access wider services [23]. The design of the previous review and its findings has been useful when planning the current review. Although it focuses on a population which is not comparable to the population of interest in this project, it has provided some understanding of the interventions in question. No specific reviews of linking interventions in early years work have been found despite extensive scoping work.

It is important to consider a number of different factors relating to linking to understand how and why it might be effective. This includes how the intervention is undertaken. This might be a very passive process such as providing information or giving out leaflets for particular services or it could be more active including a designated facilitator attending the linked service with the participant and supporting their transition to the linked service. Additionally, it will be important to consider who is involved in the process, for example is linking conducted by a healthcare professional within an existing appointment or is it undertaken by a lay health worker or someone whose role is centred on linking patients to the required services? Finally, the context in which the interaction occurs may be of significance; one element of this may be how the need for the linked service has been identified, for example is the intervention more effective when the need has been identified by the participant or by the healthcare professional? The review aims to address these questions by synthesising the available evidence on this topic.

## Aims/objectives

The primary aim of this study is to understand how health services can best help parents, carers and families with pre-school children (under the age of five) to engage with local services, groups and agencies to address their wider health and social needs. This review of the evidence will be used to inform future guidance on this topic.

The research questions, designed to answer this aim are as follows:

- Are linking schemes an effective way of engaging parents/carers/families with wider services, community groups, third sector organisations and relevant agencies?
- What are the key features of linking schemes which encourage or discourage engagement with wider resources? This includes who is involved, such as a designated facilitator, what is done and the context in which the interaction occurs.
- Is the effectiveness of linking schemes affected by how they are perceived (i.e. satisfaction with the process) by those involved in the intervention such as the practitioner or parent/carer/family?

## Methods

The protocol has been written with reference to the PRISMA-P 2015 statement for systematic review protocols, and this has been included as an Additional file 1 [27, 28]. The systematic review has also been registered with PROSPERO [29]. Methodological decisions have been guided by existing reviews or guidelines on best practice for completing systematic reviews [26, 30, 31]. The review can be described using the PICOS(S) outline (population, intervention, comparator, outcome, study design and setting), and each of these are considered in turn.

### Population

The population of interest in this review is parents, carers or families with pre-school children. This is collectively described as early years. Included within the review are expectant mothers receiving ante-natal care. No restriction has been placed on the population based on characteristics such as gender, ethnicity or socio-economic status although this information will be collected and considered within the data synthesis.

### Intervention

The intervention is defined as a process which links participants from a healthcare setting to a wider community or social service. This includes referral to voluntary organisations, services run by local authorities or other agencies. Linking between two health care settings is excluded. There is no restriction placed on the method of linking participants which may include telephone referrals, paper referrals, electronic referrals, home visits and accompanying participants to services/events. The language used to describe the intervention will not be limited as the scoping literature search suggested a range of terms are used including social prescribing, linking, referral and signposting.

### Comparator

No specific comparator will be considered in this review as many different methods of linking people with services may be included. The comparison between different methods of undertaking the intervention (including normal care/treatment as usual) will be considered as part of the data analysis and synthesis.

### Outcome measures

This review will primarily collect data against three outcomes, one primary and two secondary outcomes. The primary outcome of interest is initial engagement with the linked service, which is defined as attending the intended service on at least one occasion following linking. The first of the secondary outcomes builds on this and considers continued engagement with the linked service; this is defined as attending or engaging with the intended service on more than one occasion. Finally, measures related to satisfaction with the linking process either from the practitioner or family/parent/carer will be collected.

### Study design

All study designs will be considered for inclusion in this review as the literature on this topic is diverse, coming from a number of different fields and reported by different methods. Weighting of studies included in the final review will be based on the quality of the study, with reference to the methodological design.

As suggested by the Risk Of Bias In Systematic Reviews (ROBIS) guidelines, no specific restrictions will be placed on studies based on year of publication, language of publication or country of origin as no explicit justification for these limits can be made [32]. This is an attempt to reduce risk of bias by maintaining inclusive eligibility criteria.

### Setting

For this review, the setting of the intervention will be considered as originating in a healthcare service. This includes primary or secondary care services such as general medical services, general dental services, health visiting pathways or specialist secondary care services.

### Search strategy

The search strategy was developed first by scoping relevant literature using an internet search engine (Google Scholar); from here, papers relevant to the topic were assessed for key words and the language used to describe the intervention in question. From this, these key words were mapped against MeSH subject headings in MEDLINE and refined from there. The search uses broad descriptive headings to describe the intervention as it was recognised early on that the way in which these interventions are described is very variable. The MeSH terms used in the MEDLINE search strategy were then adapted to suit the other databases used to ensure a range of sources could be utilised.

As all study designs will be considered, filters to identify specific types of paper were not used. The search strategy was developed initially in MEDLINE then adapted for use in different databases, the search strategies are given in Additional file 2.

In addition to database searches, the reference lists of included papers will be searched to identify additional studies and a citation search will be conducted. In common with the review conducted by Mossabir et al., grey literature will form part of this review as it is expected that examples of these schemes will not be published in peer-reviewed journals but form parts of evaluation reports or other grey literature publications [26]. The search strategy for this was developed in conjunction with the subject librarian and library support team at the University of Glasgow.

### Electronic database search

A number of electronic databases in health and social sciences will be searched. The databases identified for this review are Embase, MEDLINE, CINAHL and Web of Science as they provide a wide cross section of medical and social science journals which are likely to publish on this topic. Databases will be searched from their inception to the date of the search, with no limits placed on date of publication. The reference lists of identified articles will be searched to identify any further papers, and citation searches will be performed on identified articles.

### Grey literature search

A grey literature search will be conducted to identify relevant literature on this topic not listed in the databases outlined above. A number of sources of grey literature will be used including TRiP, EThOS and Open Grey. This will be supplemented with systematic searching in an internet search engine (Google) guided by the methods outlined by Godin et al. for applying systematic search methods to grey literature [33].

### Data management

In line with current best practice advice regarding systematic reviews, all stages of the review will be completed independently by two members of the review team; this includes title and abstract screening, quality appraisal and data extraction [31]. This will consist of the corresponding author and one other member of the review team. If discrepancies are identified at any stage, these will be resolved by discussion among all members of the review team.

Records from all searches will be extracted to EndNote reference management software where duplicate records will be removed. Title and abstract screening will be conducted against the pre-agreed inclusion and exclusion criteria outlined in Additional file 3. At this stage, any papers where disagreement occurs or where insufficient information is available will be kept for full-text screening. Following this, full-text copies of all studies still under consideration for inclusion will be obtained and further assessed against the inclusion and exclusion criteria to identify the final list of publications for inclusion in the review. If multiple papers relating to a single larger study are identified, these will be considered as one single study cluster in later synthesis and analysis. Where studies are excluded, the reasons for this will be recorded in EndNote and reported in the final review.

The flow of studies through the review will be monitored using a PRISMA flow diagram and reported following the screening stage. This will immediately precede data extraction, which will be carried out using a piloted data collection form as described below.

### Data extraction

Guidance from the Cochrane Collaboration [31] and the Centre for Reviews and Dissemination [30] was used to design the data collection form which is included as Additional file 4. To ensure the appropriateness of the form and agree the final version, it will be piloted on a small number of papers and then refined to ensure maximal utility of the data extracted. Data extracted from the papers will be presented in tables. It is anticipated the tables will include descriptions of study type, study quality, intervention assessed, relevant contextual factors, study population and outcomes.

### Quality assessment and risk of bias

#### Quantitative

The Effective Public Health Practice Project Quality Assessment Tool will be used to assess the methodological quality of the included quantitative studies [34]. This is a generic tool designed to be used with a number of study designs and has been shown to have good inter-rater reliability [35, 36]. A number of domains are assessed including selection bias, study design, confounders, blinding, data collection, withdrawal, intervention integrity and analysis. A global rating for the paper classifies it as strong, moderate or weak.

#### Qualitative

It is noted in the Centre for Reviews and Dissemination (CRD) guidance that quality appraisal of qualitative studies is difficult and there is little methodological consensus in this area [30]. One of the main tools available for use in this area is the Critical Appraisal Skills Programme checklist (CASP). This has been deemed to be of equal quality to other checklists used but with easier application, consisting of 10 questions related to the rigour, credibility and relevance of the study [30, 37]. Therefore, if any studies are included which have a qualitative design, the CASP qualitative checklist will be used for quality appraisal.

### Data synthesis

Studies will be combined primarily using a narrative synthesis approach where key themes in relation to the outcomes of interest will be presented. This will include consideration of the socio-economic status and other key characteristics of the participants of the studies, if this has been reported. Quality appraisal will be used to weight the studies included in the synthesis, those studies deemed to be of higher methodological quality will be given more prominence within the synthesis and the findings of those studies with poorer methodological quality considered to provide weaker evidence. If appropriate, meta-analysis will be conducted on suitable studies. To guide the process of synthesis, frameworks provided by Popay et al. in the Economic and Social Research Council Methods Programme [38] and the CRD guidance [30] will be used. A summary of the evidence obtained in the review will be presented using tables which include descriptions of the included studies. The data will be used to illustrate the key features of successful interventions and contrast these against features seen in less successful interventions.

## Discussion

Addressing the wider determinants of health is an important focus for health services. Currently, there is a lack of evidence as to how health services can facilitate access to existing community resources and engagement activities through the process of linking individuals to services to address the wider determinants of health.

Understanding the process of linking individuals to community services from a healthcare setting may allow for more effective use of existing community resources and activities and better utilisation of existing skills within clinical teams to influence health inequalities.

The current evidence relating to linking schemes is diverse and comes from a range of different disciplines including social work, education and healthcare [21–25]. Despite there being no existing reviews relating this intervention to families with pre-school children, a previous review highlights the importance of having active involvement of healthcare practitioners in the process of linking and the essential role of facilitators for patients to access wider services [23].

It is important to consider a number of different factors relating to linking to understand how and why it might be effective. This includes how the intervention is undertaken, who is involved in the process and the context in which the interaction occurs. This review aims to address these questions by synthesising the available evidence on this topic.

## Dissemination of findings

The findings from this review will be reported as an MPH dissertation and submitted for publication in a peer-reviewed journal. Reporting will use the PRISMA statement for reporting of systematic reviews and meta-analyses (Additional file 1) [39].

## Additional files

Additional file 1: PRISMA-P checklist. (DOC 67 kb)
Additional file 2: Search strategy. (DOC 38 kb)
Additional file 3: Inclusion and exclusion criteria. (DOC 42 kb)
Additional file 4: Data extraction form. (DOCX 14 kb)

## Abbreviations

CASP: Critical Appraisal Skills Programme
CINAHL: Cumulative Index of Nursing and Allied Health Literature
CRD: Centre for Reviews and Dissemination
EMBASE: Excerpta Medica Database
MEDLINE: Medical Literature Analysis and Retrieval System Online
MeSH: Medical Subject Headings
NHS: National Health Service
NICE: National Institute for Health and Care Excellence
PICOS(S): Participants, intervention, comparator, outcomes, setting and study Design
PRISMA: Preferred Reporting Items for Systematic Reviews and Meta-Analyses
PRISMA-P: Preferred Reporting Items for Systematic Review and Meta-Analysis Protocols
PROSPERO: International Prospective Register of Systematic Reviews
