# Supplementary material for: Linking families with pre-school children from healthcare services to community resources: a systematic review protocol
Source: Syst Rev. 2017 Mar 8;6:50. doi: 10.1186/s13643-017-0417-7 (PMC5341367; doi:10.1186/s13643-017-0417-7)
Supplement: Additional file 2: — Search strategy. (DOC 38 kb) [file 13643_2017_417_MOESM2_ESM.doc]

**Additional File 2: Search Strategy**

Medline/ CINAHL

1. Exp child, preschool/
2. Exp infant/
3. Exp infant, newborn/
4. Exp family/
5. Exp parents/
6. Exp prenatal care/ or pregnancy/
7. (preschool child OR infant OR newborn OR famil* OR parent OR pregnan* OR antenatal)
8. 1 OR 2 OR 3 OR 4 OR 5 OR 6 OR 7
9. Exp referral and consultation/
10. Exp community institutional relations/
11. (referral OR signpost*)
12. 9 OR 10 OR 11
13. (social adj2 (service* or agenc* or work or welfare)).mp. (mp=title, abstract, heading word, drug trade name, original title, device manufacturer, drug manufacturer, device trade name, keyword)
14. (child* adj2 (service* or health* or care)).mp. (mp=title, abstract, heading word, drug trade name, original title, device manufacturer, drug manufacturer, device trade name, keyword)
15. ((charit* or volunt* or communit* or nonprofit) adj2 (agenc* or servic* or organisation*)).mp. (mp=title, abstract, heading word, drug trade name, original title, device manufacturer, drug manufacturer, device trade name, keyword)
16. Exp community health services/
17. Exp voluntary health agencies/
18. Exp social welfare/
19. 13 OR 14 OR 15 OR 16 OR 17 OR 18
20. 8 OR 12 OR 19

Embase

1. Exp child, preschool/
2. Exp infant/
3. Exp infant, newborn/
4. Exp family/
5. Exp parents/
6. Exp prenatal care/ or pregnancy/
7. (preschool child OR infant OR newborn OR famil* OR parent OR pregnan* OR antenatal)
8. 1 OR 2 OR 3 OR 4 OR 5 OR 6 OR 7
9. Exp patient referral/
10. Exp public relations/
11. (referral OR signpost*)
12. 9 OR 10 OR 11
13. (social adj2 (service* or agenc* or work or welfare)).mp. (mp=title, abstract, heading word, drug trade name, original title, device manufacturer, drug manufacturer, device trade name, keyword)
14. (child* adj2 (service* or health* or care)).mp. (mp=title, abstract, heading word, drug trade name, original title, device manufacturer, drug manufacturer, device trade name, keyword)
15. ((charit* or volunt* or communit* or nonprofit) adj2 (agenc* or servic* or organisation*)).mp. (mp=title, abstract, heading word, drug trade name, original title, device manufacturer, drug manufacturer, device trade name, keyword)
16. Exp community health services/
17. Exp voluntary health agencies/
18. Exp social welfare/
19. 13 OR 14 OR 15 OR 16 OR 17 OR 18
20. 8 OR 12 OR 19

Web of Science (Core Collection)

1. Topic: (child)
2. Topic: (infant)
3. Topic: (baby)
4. Topic: (newborn)
5. Topic: (preschool)
6. Topic: (famil*)
7. Topic: (parent*)
8. Topic: (pregnan*)
9. #8 OR #7 OR #6 OR #5 OR #4 OR #3 OR #2 OR #1
10. Topic: ((charit* OR volunt* OR communit* OR nonprofit) NEAR/2 (agenc* OR service* OR organisation*))
11. Topic: ((child*) NEAR/2 (service* OR agenc* OR health* OR care))
12. Topic: (social NEAR/2 (service* OR agenc* OR work OR welfare OR care))
13. #12 OR #11 OR ‘10
14. Topic: refer*
15. Topic: (case NEAR/2 management)
16. #14 OR#15
17. #9 AND #13 AND #16
