# Supplementary material for: Linking families with pre-school children from healthcare services to community resources: a systematic review protocol
Source: Syst Rev. 2017 Mar 8;6:50. doi: 10.1186/s13643-017-0417-7 (PMC5341367; doi:10.1186/s13643-017-0417-7)
Supplement: Additional file 3: — Inclusion and exclusion criteria. (DOC 42 kb) [file 13643_2017_417_MOESM3_ESM.doc]

**Additional File 3: Agreed inclusion and exclusion criteria**

Screening Criteria- title and abstract

| Inclusion | Exclusion |
| --- | --- |
| The intervention described must have some form of linking component and must originate in a healthcare setting  The paper must report at least one of the outcomes of interest (primary engagement, secondary engagement, satisfaction) in relation to the intervention  The paper may be of any study design or methodology  The paper may be of any language  The paper may be from any country of origin  Papers should be related to children, infants, babies, families or parents  Sure Start as an identified programme should be included  Interventions relating to parenting, supporting families/parents/children, improving access or addressing (wider) needs should be included although this terminology may not be exhaustive and other terms may be appropriate  Referral is a key term which should be identified  Other terms which would be of interest to include are collaboration, case management, inter-professional working and integration  Any papers where the topic is unclear should be kept for further screening until it can be properly assessed.  Community-based interventions/initiatives should be included if they fulfil the other inclusion criteria  Papers related to the third sector or voluntary organisations should be included | Papers should not focus on adults, older adults or adolescents, unless the adolescent is receiving maternity care  Papers where the referral is from health care to health care should be excluded  Papers where the source of the referral is unclear but the final destination is health care (*i.e.,* referral to specialist secondary care) should be excluded  Papers where the topic is treatment of specific conditions in health care should be excluded |

Inclusion and Exclusion Criteria- Full text screening

| Inclusion | Exclusion |
| --- | --- |
| Any study design or methodology  No language restrictions  No restriction on country of origin  Population of interest must be pre-school (age under 5) children or their parents/carers  The intervention described must have some form of linking component  Linking intervention must originate in healthcare setting  The paper must report at least one of the outcomes of interest (primary engagement, secondary engagement, satisfaction) in relation to the intervention | Papers should not focus on adults, older adults or adolescents, unless the adolescent is receiving maternity care  Papers where the referral is from health care to health care should be excluded  Papers where the source of the referral is unclear but the final destination is health care (*i.e.,* referral to specialist secondary care) should be excluded  If the paper does not report against the outcomes of interest it should be excluded. |
