# Supplementary material for: Linking families with pre-school children from healthcare services to community resources: a systematic review protocol
Source: Syst Rev. 2017 Mar 8;6:50. doi: 10.1186/s13643-017-0417-7 (PMC5341367; doi:10.1186/s13643-017-0417-7)
Supplement: Additional file 4: — Data extraction form. (DOCX 14 kb) [file 13643_2017_417_MOESM4_ESM.docx]

**Additional File 4: Data Extraction Form**

| **General Information** | | | | |
| --- | --- | --- | --- | --- |
| Person Completing Form: | | | Date: | |
| Record Number: | | | Author: | |
| Article Title: | | | | |
| Citation: | | | | |
| **Study Characteristics** | | | | |
| Study Design: | | | | |
| Study Dates: | | | | |
| Aim/Objective of study: | | | | |
| **Target Population** | | | | |
| Age: | Gender: | | | Ethnicity: |
| Socio-Economic Status: | | | Other relevant details : | |
| **Setting** | | | | |
| Country where study conducted:  Setting: | | | | |
| Sample Size | | | | |
| No of participants | | No of participants in final analysis | | |
| No of participants lost to follow up, withdrew, excluded | | | | |

| **Intervention** | |
| --- | --- |
| Description of intervention  (including details of who is involved, what is done) | |
| Comparator? | |
| **Outcome Data** | |
| Primary outcome- Primary Engagement | |
| Reported? | Definition |
| Yes / No / Partial |  |
| Measured by? | Result |
|  |  |
| Secondary Outcome- Sustained engagement | |
| Reported? | Definition |
| Yes / No / Partial |  |
| Measured by? | Result |
|  |  |
| Secondary Outcome- Satisfaction Measures | |
| Reported? | Definition |
| Yes / No / Partial |  |
| Measured by? | Result/ Description |
|  |  |
| Notes: | |
|  | |
